# Rapid Fabricating Technique for Multi-Layered Human Hepatic Cell Sheets by Forceful Contraction of the Fibroblast Monolayer

**DOI:** 10.1371/journal.pone.0070970

**Published:** 2013-07-26

**Authors:** Yusuke Sakai, Makiko Koike, Hideko Hasegawa, Kosho Yamanouchi, Akihiko Soyama, Mitsuhisa Takatsuki, Tamotsu Kuroki, Kazuo Ohashi, Teruo Okano, Susumu Eguchi

**Affiliations:** 1 Department of Surgery, Nagasaki University Graduate School of Biomedical Sciences, Nagasaki, Japan; 2 Institute of Advanced Biomedical Engineering and Science, Tokyo Women's Medical University, Tokyo, Japan; University of California, Merced, United States of America

## Abstract

Cell sheet engineering is attracting attention from investigators in various fields, from basic research scientists to clinicians focused on regenerative medicine. However, hepatocytes have a limited proliferation potential *in vitro*, and it generally takes a several days to form a sheet morphology and multi-layered sheets. We herein report our rapid and efficient technique for generating multi-layered human hepatic cell (HepaRG® cell) sheets using pre-cultured fibroblast monolayers derived from human skin (TIG-118 cells) as a feeder layer on a temperature-responsive culture dish. Multi-layered TIG-118/HepaRG cell sheets with a thick morphology were harvested on day 4 of culturing HepaRG cells by forceful contraction of the TIG-118 cells, and the resulting sheet could be easily handled. In addition, the human albumin and alpha 1-antitrypsin synthesis activities of TIG-118/HepaRG cells were approximately 1.2 and 1.3 times higher than those of HepaRG cells, respectively. Therefore, this technique is considered to be a promising modality for rapidly fabricating multi-layered human hepatocyte sheets from cells with limited proliferation potential, and the engineered cell sheet could be used for cell transplantation with highly specific functions.

## Introduction

Engineered cell sheets composed of cells and cell-produced extracellular matrix (ECM) have become attractive for researchers from various fields ranging from basic science to clinical applications for regenerative medicine. Cell sheets have several advantages compared with biomaterials, such as bacterial cellulose and polymers, in the clinical field; abundant cell-cell and cell-ECM adhesions and high viability *in vivo*. Many researchers have already reported *in vitro* investigations of multi-layered cell sheets, such as fabrication methods using a temperature-responsive culture dish (TRCD) and a gelatin stamp or magnetic force system with a high cell density, vascularized sheet tissues using endothelial cells for the biomimesis and maintenance of cell survival, and cell distributions based on biology and computational chemistry [1]–[4]. Cultured cell-derived tissues, including a multi-layered cell sheet formed by either the proliferation of cells or layer-by-layer deposition, could be transplanted and engrafted easily, because they have dramatically improved the handling compared to the single-layered thin cell sheets. In addition, several medical applications of cell sheets have already been reported. For example, patients with esophageal stenosis after endoscopic submucosal dissection (ESD) and severe corneal opacification were treated by the application of oral mucosal epithelium cell sheets containing epithelial stem cells [5], [6]. Sawa et al. [7] treated patients with dilated cardiomyopathy (DCM) using myoblast sheets. Therefore, the fabrication of multi-layered cell sheets is one of the hottest topics related to cell sheet engineering.

Hepatocyte sheets were also strongly anticipated for various clinical applications. Several researchers reported that single- and multi-layered rat and mouse primary hepatocyte sheets could be fabricated by using a TRCD, a special substrate with electrochemical desorption of a self-assembled monolayer (SAM) of alkanethiol, and a bioreactor [8]–[10]. In addition, endothelial cell sheets were co-cultured with hepatocyte sheets to maintain the liver-specific functions of hepatocytes [11], [12]. However, primary hepatocytes, which have limited proliferation potential *in vitro*, are difficult to form a sheet morphology and multi-layered artificial tissues, because cell sheets typically require the formation of a confluent monolayer through cell proliferation or inoculation at a high density of cells. In fact, the previous papers showed that a minimum of three days was required for the formation of a confluent monolayer of hepatocytes [7], [9], [10]. There have been no reports of a rapid and efficient technique for fabricating multi-layered hepatocyte sheets *in vitro* to improve the maintenance of the higher functions of the tissues and to allow for more mass production of transplantable hepatocyte sheets.

In this study, we focused on the forceful contraction of fibroblasts when they formed cell sheets, and established a new method for the rapid and efficient fabrication of multi-layered human hepatic cell sheets without the need for layer-by-layer deposition and/or cell proliferation. Furthermore, the thickness and liver-specific functions of the hepatic cell sheets were evaluated to elucidate their characteristics and the advantages of this fabrication technique. The goals of this study were to establish a rapid fabrication technique for multi-layered cell sheets with good handling and highly specific functions using cells with a limited proliferation potential or high contact inhibition, including primary hepatocytes, pancreatic islet cells and fibroblasts for cell transplantation.

## Materials and Methods

### HepaRG Cells

HepaRG® cells (HRP116; Biopredic International, Rennes, France) are terminally well-differentiated hepatic cells derived from a human liver progenitor cell line and have limited proliferation potential (almost no growth according to the product specification) [13]. The HepaRG cell suspension was prepared from cryopreserved vials immediately after thawing, and were cultured in the basal medium for HepaRG cells (Medium670; previously supplemented with 10% fetal bovine serum (FBS) and 0.5% dimethyl sulfoxide (DMSO); Biopredic International) supplemented with 2 mM l-glutamine, 100 U/mL penicillin and 100 µg/mL streptomycin (all from Invitrogen, Carlsbad, CA, USA).

### TIG-118 Cells

TIG-118 cells (JCRB0535; Health Science Research Resources, Osaka, Japan), which are fibroblasts derived from human skin, were cultured as a continuous monolayer in a 90 mm tissue culture dish (Nalgene Nunc International, Rochester, NY, USA) containing 10 mL of Minimum Essential Medium (MEM) supplemented with 10% FBS, 2 mM l-glutamine, 100 U/mL penicillin and 100 µg/mL streptomycin. The TIG-118 cell suspension was obtained by treating the 90% confluent monolayers formed on a tissue culture dish with 0.25% trypsin-EDTA (all from Invitrogen).

### Fabrication Process for the TIG-118/HepaRG Cell Sheets

**Figure 1:**
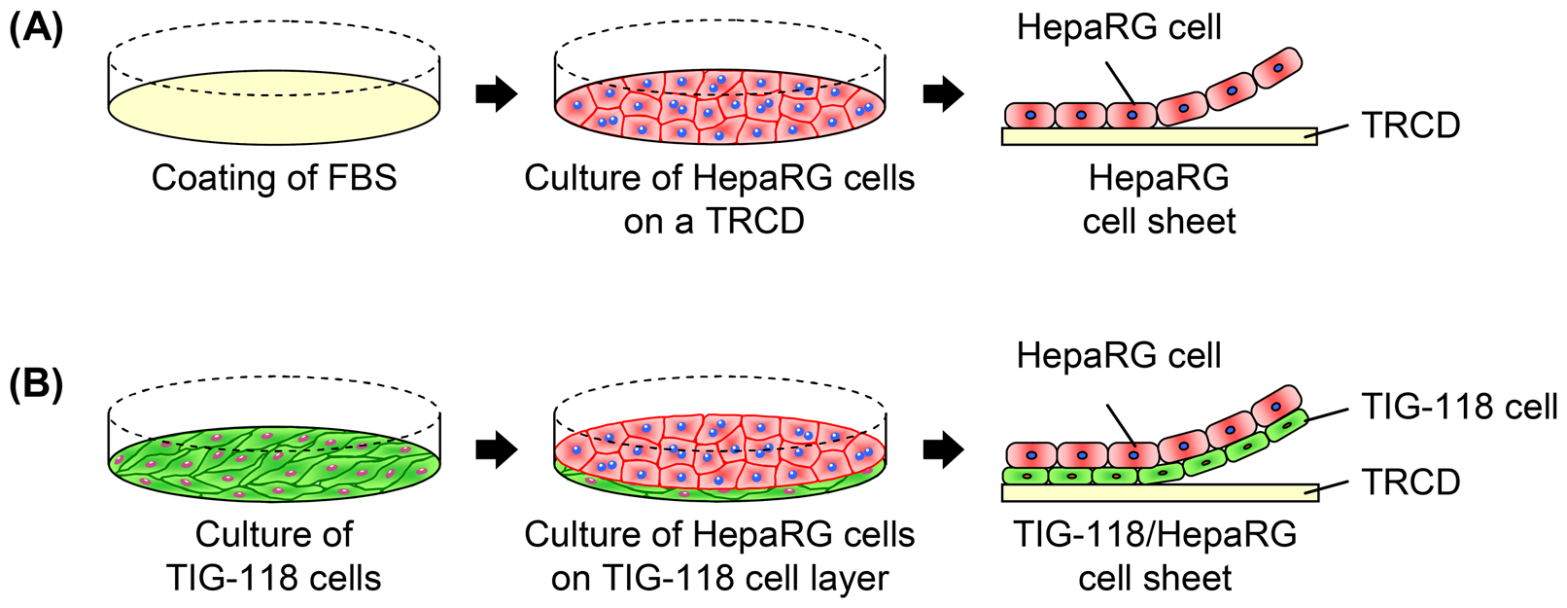
Schematic diagrams of the fabrication process used for the human hepatic cell (HepaRG) sheets. The processes used to fabricate the HepaRG cell-only sheet (A) and TIG-118/HepaRG cell sheet (B).

Figure 1 shows schematics of the fabrication process for two types of the hepatic cell sheets. Fig. 1A shows the fabrication process using only HepaRG cells as a control. Before seeding the HepaRG cells, the surface of a 35 mm TRCD (UpCell®; CellSeed Inc., Tokyo, Japan) was coated with 0.5 mL FBS overnight to promote cell adhesion. A HepaRG cell suspension was then inoculated onto the TRCD at a density of 1.4×10^5^ cells/cm^2^. Fig. 1B shows the process of fabricating a TIG-118/HepaRG cell sheet. A TIG-118 cell suspension was inoculated onto a TRCD at a density of 2.3×10^4^ cells/cm^2^, and cultured in MEM. After the TIG-118 cells formed a confluent monolayer within three days of culture, a HepaRG cell suspension was inoculated at a density of 1.4×10^5^ cells/cm^2^.

After one and four days of culturing the HepaRG cells, the cells were incubated at 20°C. These cells were harvested as cell sheets by gently pipetting from the edge of the dish. All the cells were cultured in a basal medium under a humidified atmosphere of 5% CO_2_ and 95% air at 37°C. Two milliliters of culture medium were changed 24 h after the inoculation, and every two days thereafter.

### Evaluation of Cell Morphologies and Distributions

To evaluate the cellular morphologies and distributions of TIG-118 cells and HepaRG cells, CellTracker™ Green CMFDA and Orange CMRA (Molecular Probes, Eugene, OR, USA) were used to identify TIG-118 cells and HepaRG cells, respectively. The staining solutions of 5 µM CellTracker Green and 10 µM CellTracker Orange were prepared by adding 0.5 and 1.0 µL of 10 mM CellTracker stock solution in DMSO to 1 mL of Dulbecco’s modified Eagle’s medium (DMEM; Wako Pure Chemical Industries Ltd., Osaka, Japan) supplemented with 2 mM l-glutamine, 100 U/mL penicillin, and 100 µg/mL streptomycin, respectively. The cultured confluent TIG-118 cells on a TRCD were stained using 5 µM CellTracker Green solution for 20 min at 37°C. To stain the HepaRG cells, a HepaRG cell suspension was incubated in the 10 µM CellTracker Orange solution for 20 min at 37°C before inoculation. After one and three days of culturing HepaRG cells, fluorescence of the cells on a TRCD was detected by using a confocal laser scanning microscope (FV10i; Olympus, Tokyo, Japan). To quantitatively analyze the cell population rate, the areas of green and red were assessed from the captured fluorescent images of every 1-µm horizontal section.

After four days of culturing HepaRG cells, the cell sheets were fixed with 4% paraformaldehyde phosphate buffer solution (PFA; Wako) for 10 min. The fixed cell sheets were permeabilized with 0.1% Triton-X for 5 min and blocked in 1% bovine serum albumin (BSA) solution for 20 min. They were then incubated with PBS buffer containing 5 unit/mL Alexa Fluor 568 Phalloidin (Molecular Probes) and 1% BSA for 20 min to stain them for F-actin, and then were incubated with PBS containing 1 µg/mL 4′,6-diamidiono-2-phenylindole (DAPI; DOJINDO, Kumamoto, Japan) for 5 min to stain the nuclei in the cells. Stained cell sheets were mounted with ProLong gold antifade mounting medium (Molecular Probes). The double- and triple-stained cell sheets were assessed for cross-sectional distributions and for their morphology using a confocal laser scanning microscope. All procedures were performed at room temperature.

### Ultrastructural Analysis

After four days of culture, the TIG-118/HepaRG cell sheets were fixed with 2.5% glutaraldehyde in 0.1 M phosphate buffer (pH 7.4). The samples were fixed in phosphate-buffered 1% osmium tetroxide for 2 h at 4°C, followed by dehydration through a graded ethanol series. The dehydrated samples were embedded in Epon 812 (TAAB Laboratories Equipment Ltd., Berkshire, England). Ultrathin sections were cut with an ultramicrotome (Ultracut S, Leica, Austria) with a diamond knife and double-stained with uranyl acetate and lead nitrate, then observed with an electron microscope (JEM-1200EX, JEOL, Tokyo, Japan) at an accelerating voltage of 60 kV.

### Evaluation of Cell Sheet Diameter and Thickness

Two types of hepatic cell sheets were generated after four days of culturing the HepaRG cells, and the TIG-118 cell-only sheet was made after seven to nine days of culturing the TIG-118 cells. The cell sheet diameters and thicknesses were evaluated and compared. The exterior photographs of cell sheets were captured to evaluate the cell sheet diameters after approximately 30 minutes of cell sheet fabrication when contraction of the cell sheets had almost stopped. The diameters of cell sheets were calculated as the ratio of the cell sheet area to the original TRCD culture surface area. To evaluate the thicknesses of the cell sheets, cross-sectional images of the cell sheets were evaluated. The cell sheets fixed with 4% PFA were embedded with paraffin, then the paraffin-embedded sections were stained by hematoxylin and eosin (HE). The stained cross-section images were captured using an optical microscope (BX53; Olympus, Tokyo, Japan). These areas and thicknesses were measured using the NIS-Elements software program (Nikon, Tokyo, Japan).

### Cell Viability Assay

Calcein-AM and propidium iodide (PI) were used to identify live and dead cells, respectively (Cellstain Double Staining Kit; DOJINDO, Kumamoto, Japan). The assay solution mixing calcein-AM and PI was prepared by adding 2 µL of 1 M calcein-AM stock solution and 3 µL of 1.5 M PI solution to 1 mL of Dulbecco’s modified Eagle’s medium supplemented with 2 mM of l-glutamine, 100 U/mL of penicillin and 100 µg/mL of streptomycin. The cells on TRCD and the cell sheets after two and 24 hours of reculturing on a glass-based dish (IWAKI, Asahi Glass Co., LTD., Tokyo, Japan) were incubated with the assay solution for 15 minutes and then washed with PBS solution. The double-stained cells were assessed using a fluorescence microscope (Eclipse Ti-U; Nikon).

### Evaluation of Liver-specific Functions

The human albumin and alpha 1-antitrypsin (A1AT) synthesis activities were evaluated under both culture conditions between one and three days of culturing HepaRG cells. The concentrations of human albumin and A1AT in the culture medium were determined by enzyme-linked immunosorbent assays (ELISAs). Rabbit anti-human albumin (6 µg/mL) and horseradish peroxidase (HRP)-conjugated goat anti-human albumin (10 µg/mL) antibodies were used to detect human albumin (both antibodies were purchased from MP Biomedicals, LLC-Cappel products, Irvine, CA, USA). Goat anti-human A1AT (5 µg/mL; Bethyl Laboratories, Inc., Montgomery, Texas, USA) and HRP-conjugated goat anti-human A1AT (7 µg/mL; Fitzgerald Industries International, Inc., Concord, MA, USA) antibodies were used to detect human A1AT.

### Statistical Analysis

The data are presented as the means ± standard deviation (SD) of at least five time points from two independent cell preparations. The statistical analysis was performed for the numerical variables by using a repeated-measures analysis of variance (ANOVA; diameter and thickness) and Mann-Whitney’s *U*-test (liver-specific functions), with values of **P<0.01 and *P<0.05 considered to be statistically significant.

## Results and Discussion

### HepaRG Cell Morphology on TIG-118 Cell Monolayers

**Figure 2:**
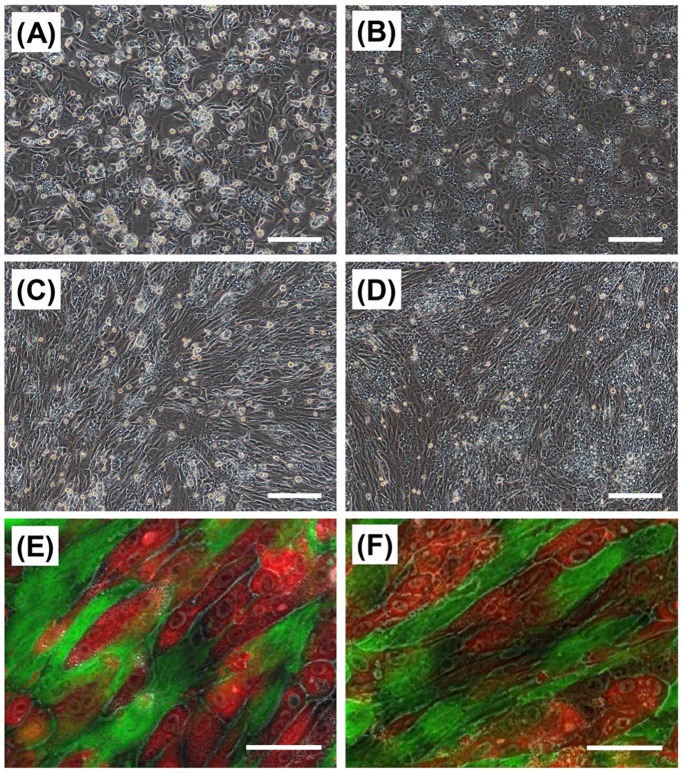
Cell morphologies of HepaRG cells and TIG-118/HepaRG cells on TRCD. Phase-contrast (A–D) and fluorescent (E, F) micrographs of the HepaRG cells (A, B) and TIG-118/HepaRG cells (C–F) on a TRCD. After one day (A, C, E) and three days (B, D, F) of culturing HepaRG cells. Green (CellTracker Green CMFDA): TIG-118 cells, Red (CellTracker Orange CMRA): HepaRG cells. The bars represent 200 µm (A–D) and 50 µm (E, F).

The HepaRG cells adhered and spread on the FBS-coated TRCD on day 1 of culture, and some of the HepaRG cells had a cuboidal cell morphology (Fig. 2A). The HepaRG cells had a liver-specific multiangular shape and formed a confluent monolayer within three days of culture (Fig. 2B). Approximately half of the HepaRG cells had a colony-like morphology at the location of high cell density, while the others formed a thin monolayer. In contrast, most of the HepaRG cells quickly adhered onto the TIG-118 cell monolayers compared to the FBS-coated TRCD. Each of HepaRG cells on the TIG-118 cells showed an extended spindle-like morphology along the shape of the TIG-118 cells (Figs. 2C and 2E). It has been reported that the morphology and the functional expression levels of hepatocytes and stem cells are subject to anchorage-dependent control, and the HepaRG cell morphology on the TIG-118 cells was similar to that on a linear cell adhesion patterning substrate [14]–[16]. Within three days of culturing HepaRG cells, nearly all hepatic cell colonies surrounded by TIG-118 cells showed three-dimensional (3D) morphology and cord-like colony formation due to the limited space for extension (Figs. 2D and 2F). This cell morphology is expected to improve and maintain the hepatocyte functions of the cells during long-term culture, as is the case with 3D small islands of rat primary hepatocytes that are surrounded by fibroblasts in the patterning co-culture system [17]–[20].

### Distributions of HepaRG and TIG-118 Cells on TRCD

**Figure 3:**
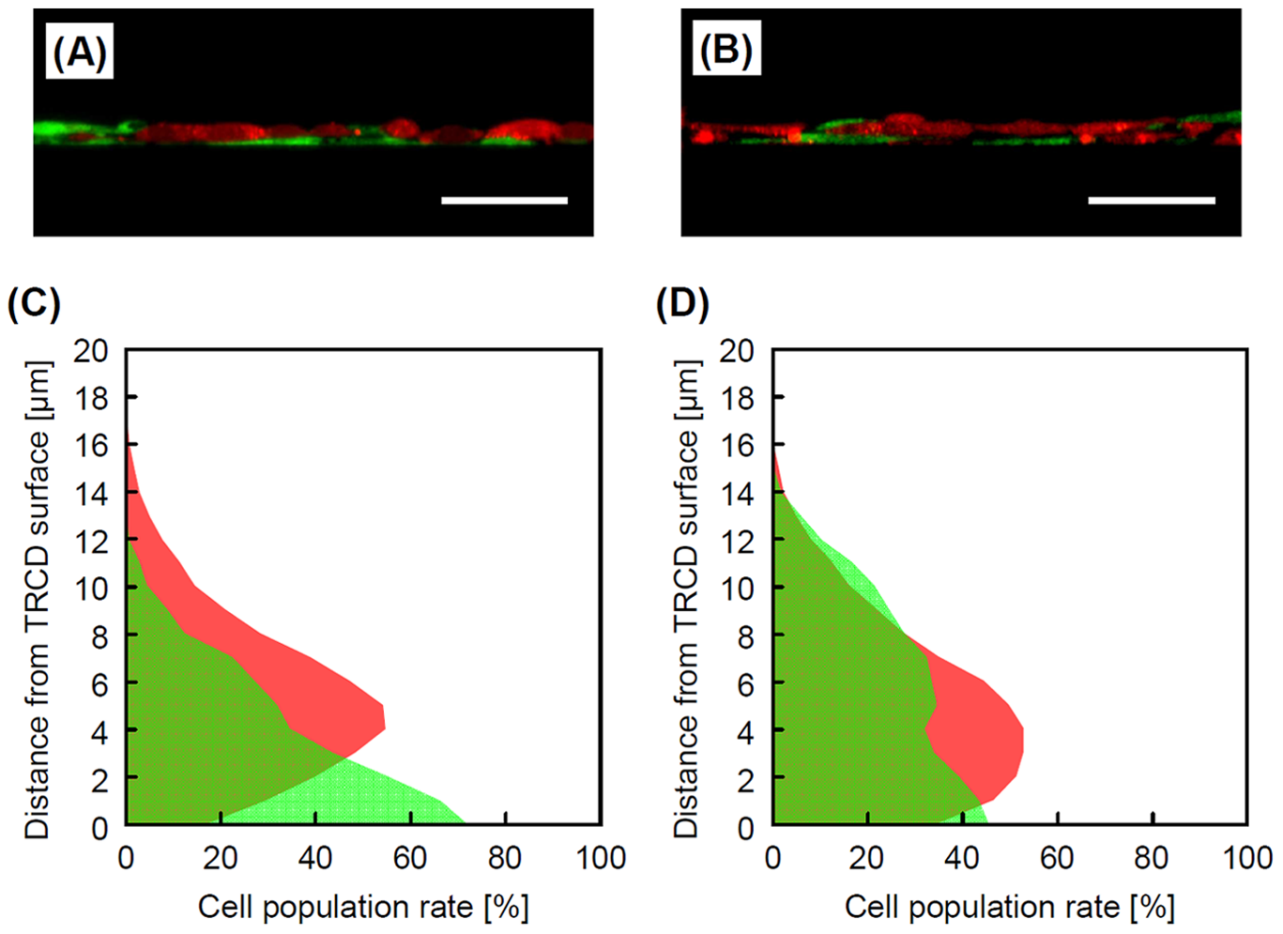
Cell distributions of TIG-118/HepaRG cells on TRCD. Cross-section views (A, B) and the cell population (C, D) of the TIG-118/HepaRG cells. After one day (A, C) and three days (B, D) of culturing HepaRG cells. The bars represent 50 µm.

Figure 3 shows the cross-section distributions and cell population rates of HepaRG cells and TIG-118 cells. The HepaRG cells adhered onto the thin TIG-118 cell monolayers after one day of culturing HepaRG cells (Fig. 3A). Within three days of culturing HepaRG cells, some TIG-118 cells had migrated from the bottom to the top, and sandwiched the HepaRG cells (Fig. 3B). It seems that some HepaRG cells pushed away the TIG-118 cells, which extended in thin layers in the initial stage of culture, and they adhered directly onto the TRCD. In addition, the TIG-118 cells which were pushed away adhered and extended on HepaRG cells due to cell migration. To demonstrate the migration of cells, we evaluated the cell population rates in every 1-µm horizontal section on days 1 and 3 of culture (Figs. 3C and 3D). Many TIG-118 cells adhered to the bottom face of TRCD on day 1 of the culture. On day 3 of the culture, the population of TIG-118 cells decreased on the bottom face and migrated to the top of the cell sheet compared with that observed on day 1 of the culture. It is known that cell migration occurs in relation to the degree of adhesion and interaction between cells and/or between cells and the ECM [3], [21], [22]. Additional interactions between different types of cells and/or between the cell and the culture substrate will also contribute to such specific cell distributions.

### Rapid Fabrication of TIG-118/HepaRG Cell Sheets

**Figure 4:**
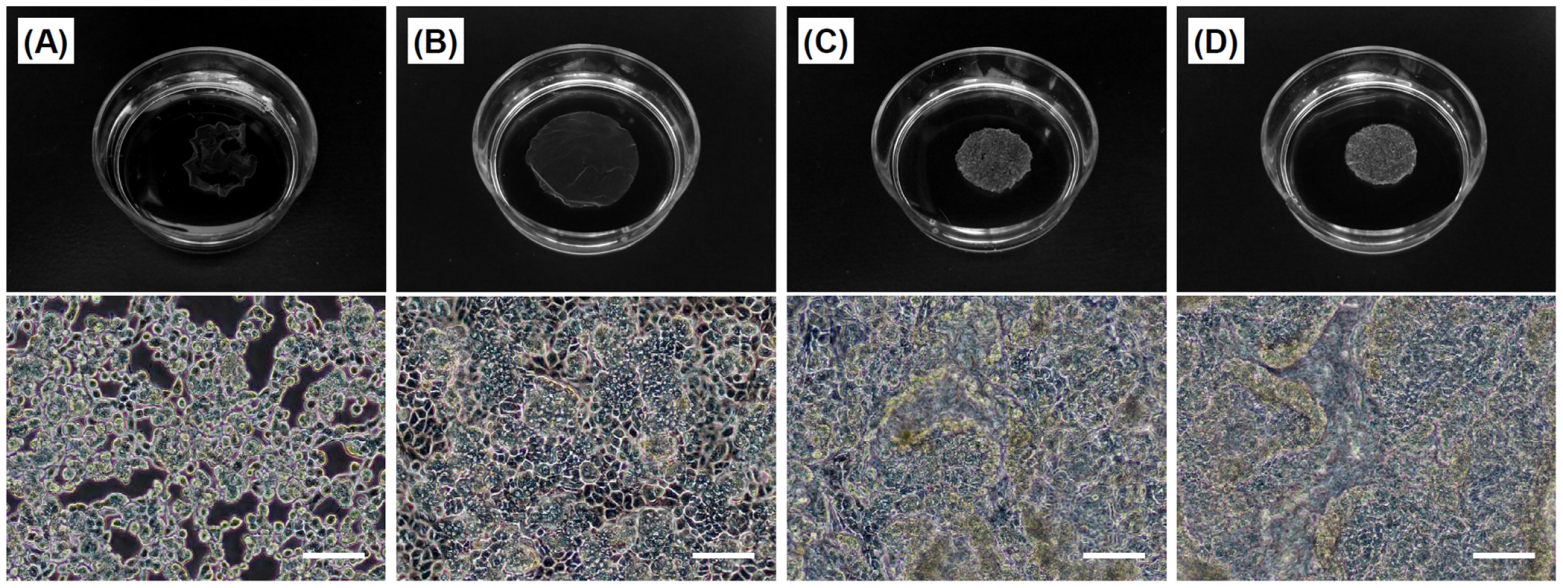
Cell morphologies of HepaRG cell and TIG-118/HepaRG cell sheets. Exterior photographs (above) and phase-contrast micrographs (below) after incubation at 20°C. (A, B) HepaRG cells and (C, D) TIG-118/HepaRG cells. After one day (24 hours) (A, C) and four days (B, D) of culturing HepaRG cells. The bars represent 100 µm.

As primary hepatocytes rarely grow under culture conditions, rapid cell sheet formation is very difficult and the functions of the hepatocytes decrease markedly over the course of culture [23], [24]. In fact, the HepaRG cells were also unable to form complete cell sheets on day 1 (24 hours) of culture; the sheet-like cell aggregates had many holes and exhibited low intensity for handling (Fig. 4A). However, the rapid and stable fabrication of hepatocyte sheets leads to the maintenance of the higher functions and better mass production of transplantable hepatocyte sheets, while keeping the fabrication process simple. Therefore, we think that this rapid fabrication system using fibroblasts with forceful contraction as feeder cells is an effective approach for cell sheet engineering. For this procedure, TIG-118/HepaRG cell sheets were harvested after incubation at 20°C for 2 hours after one day (24 hours) of culturing HepaRG cells (Fig. 4C). Both of the culture conditions allowed for the formation of cell sheets on day 4 of culturing HepaRG cells (approximately 4 and 2 hours of incubation at 20°C under mono-culture and co-culture conditions, respectively) (Figs. 4B and 4D). The detachment of hepatic cell sheets will be promoted by the pull strength of the TIG-118 cells due to the power of their forceful construction. In contrast, only HepaRG cells exhibited little power for detachment.

Ito *et al.* [2] reported that multi-layered NIH 3T3/HepG2 cell sheets can be fabricated within one day of culturing HepG2 cells using a magnetic force system. However, in that study, the HepG2 cells had a high potential for proliferation and were cultured at approximately a 4-fold higher level of cell density compared with our system. In this paper, the HepaRG cells had a limited potential for proliferation and were cultured at a low density, as observed in the primary hepatocyte cultures. Therefore, our co-culture system is a new technique that can be used to rapidly fabricate hepatic cell sheets with a low potential for proliferation.

### Cell Morphology and Ultrastructure of the Multi-layered TIG-118/HepaRG Sheets

**Figure 5:**
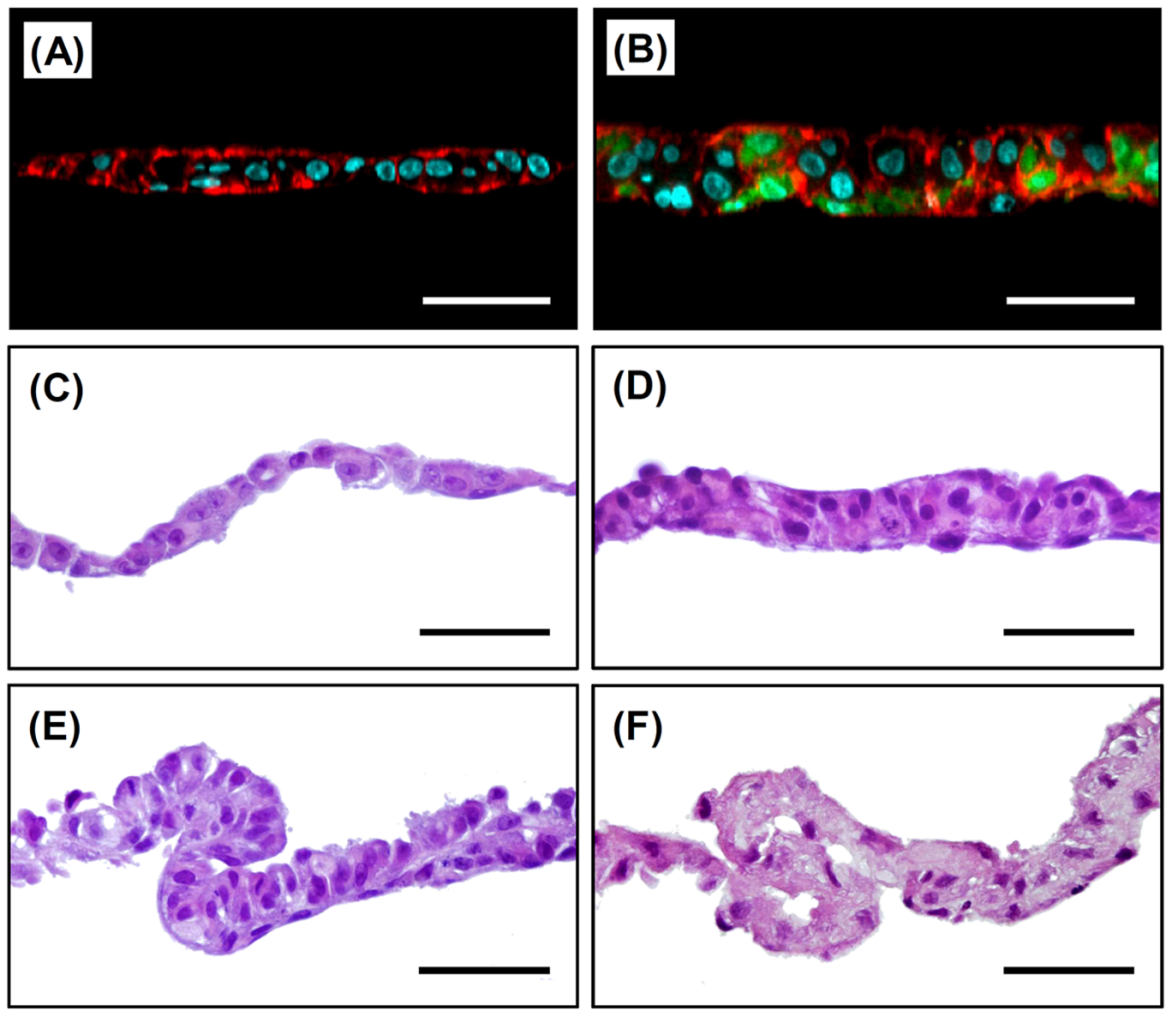
Cell distributions and HE stained images of the cross-sections of cell sheets. Fluorescent (A, B) and HE (C–F) stained images. (A, C) HepaRG cells, (B, D, E) TIG-118/HepaRG cells and (F) TIG-118 cells. Green (CellTracker Green CMFDA): TIG-118 cells, Red (Alexa Fluor 568 Phalloidin): F-actin, Blue (DAPI): nucleus. The bars represent 50 µm.

Figure 5A–E show the fluorescent and HE-stained images of cross-sections of cell sheets after four days of culturing HepaRG cells. The HepaRG cell sheets, which were formed from only HepaRG cells, consisted of almost all single-layered cells with an aspect ratio of nearly one despite the presence of a mixture of 3D colonies and extended cells (Figs. 5A and 5C). The HepaRG cells formed uniform cell sheets without wrinkles even after shrinking (Fig. 4B and 5C). In contrast, the TIG-118/HepaRG cells formed multi-layered (double- to triple-layered) cell sheets consisting of HepaRG cells with ball-like and/or vertically elongated cell morphology (Figs. 5B and 5D). Many TIG-118 cells consisted of the bottom part of cell sheets, however, some TIG-118 cells presented in the middle and top of cell sheets. Laminate adhesion between the cell sheets was also observed in some places (Fig. 5E), which appeared macroscopically to be “wrinkles” (Fig. 4D). These phenomena are believed to be attributable to the significant contraction of the very thinly extended TIG-118 cell layers which developed a cuboidal-shaped and the strong adhesion between fibroblasts. In fact, laminate adhesion and wrinkles were observed in the TIG-118 cell-only sheet (Fig. 5F).

Green *et al.* [25], [26] and Nishida *et al.* [5] reported a method using a feeder layer of 3T3 cells for the formation of epidermal keratinocyte and oral mucosal epithelium cell sheets to promote cell proliferation for the formation of multi-layered cell sheets. In addition, several researchers reported that multi-layer cell sheets could be fabricated by the deposition of cell sheets one-by-one [1]–[4], [8], [11], [12]. On the other hand, our technique focused on the forceful contraction of fibroblasts was able to rapidly and easily fabricate multi-layered human hepatic cell sheets without requiring layer-by-layer deposition or cell proliferation; the change in the cell density of TIG-118/HepaRG cells was no more than 1.1-fold increase during the two days of culture (day 1 to day 3) (data not shown). This limited proliferation is due to the high level of differentiation of the HepaRG cells, the cell contact inhibition of the fibroblasts and the DMSO added in the culture medium.

**Figure 6:**
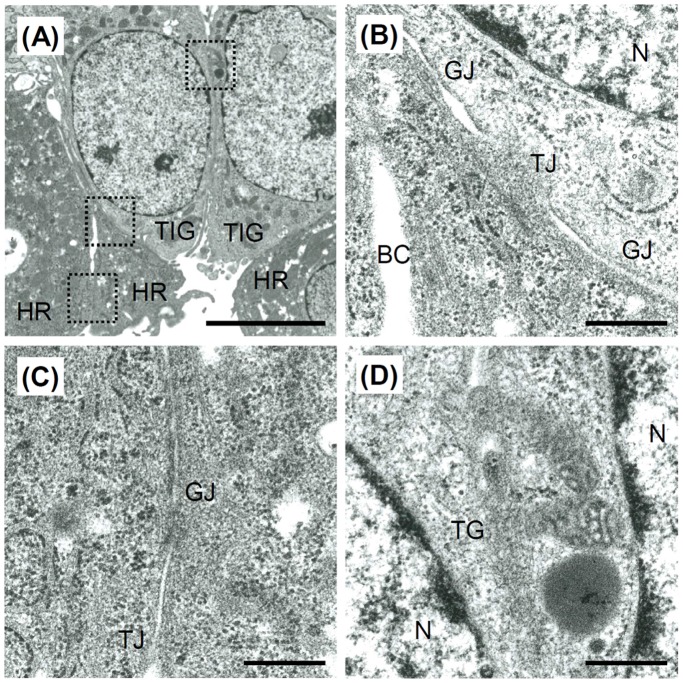
Ultrastructures of the TIG-118/HepaRG cell sheets observed by TEM. (A) Low magnification and (B–D) high magnification images of the cell-cell adhesion between HepaRG cell and TIG-118 cell (B), HepaRG cells (C) and TIG-118 cells (D). HR, HepaRG cell; TIG, TIG-118 cell; N, nucleus; BC, bile canaliculi; TG, tight junctions; GJ, gap junctions. The bars represent 5 µm (A) and 500 nm (B–D).

Figure 6 shows the ultrastructures of cross-sections of the TIG-118/HepaRG cell sheets after four days of culture. HepaRG cells that adhered to the TIG-118 cells formed tight junctions and gap junctions (Fig. 6B). In addition, HepaRG cells formed bile canals (Fig. 6B), tight junctions and gap junctions (Fig. 6C). The TIG-118 cells also formed a long tight junctional region (Fig. 6D). These abundant cell-cell adhesions, including those between HepaRG cells and TIG-118 cells, allowed for the rapid formation of cell sheets after a short culture period.

### Diameter and Thickness of Cell Sheets

**Figure 7:**
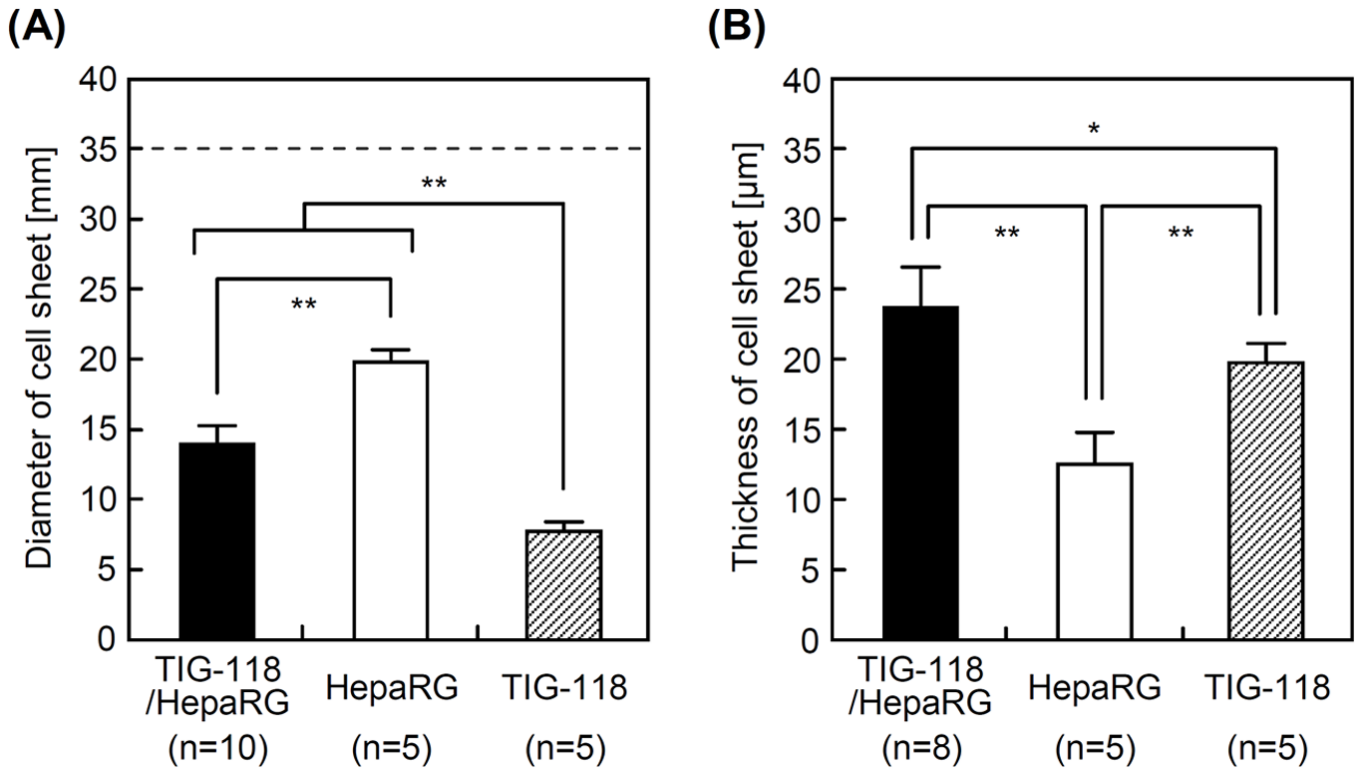
Characteristics of cell sheets. Diameters (A) and thicknesses (B) of cell sheets. The dashed line indicates the diameter of a TRCD.

The diameters of the cell sheets are shown in Fig. 7A. The area of the cell sheets consisted of TIG-118/HepaRG cells, only HepaRG cells and only TIG-118 cells contracted to 19.5±2.6% (14.0±1.4 mm in diameter), 32.4±2.6% (19.9±0.8 mm in diameter) and 4.9±0.8% (7.7±0.6 mm in diameter) of the original TRCD culture surface area, respectively. A forceful contraction of the TIG-118 cells was observed in the TIG-118 cell culture conditions. Furthermore, the diameter of the TIG-118/HepaRG cell sheet was smaller than that of the HepaRG cell-only sheet, despite the fact that the TIG-118/HepaRG cell sheet consisted of many cells compared with the HepaRG cell-only sheet. This result was due to the forceful contraction of the TIG-118 cells, because the TIG-118 cells were extended as a thin layer on the TRCD surface.

The thicknesses of TIG-118/HepaRG cell, HepaRG cell-only and TIG-118 cell-only sheets (excluding the laminated area between the sheets of TIG-118 cells) were 23.8±2.8 µm, 12.6±2.2 and 19.8±1.3 µm, respectively (Fig. 7B). The thickness of the hepatic cell sheets correlated inversely with the area. One reason for the increased thickness of the TIG-118/HepaRG cell sheet is that the sheet was layered with a TIG-118 cell layer. The forceful contraction described above was also a strong contributor to the increased thickness. Our newly developed system is a remarkably simpler and more rapid method for fabricating multi-layered thick cell sheets compared with those in the previous reports because it does not require layer-by-layer deposition or cell proliferation.

**Figure 8:**
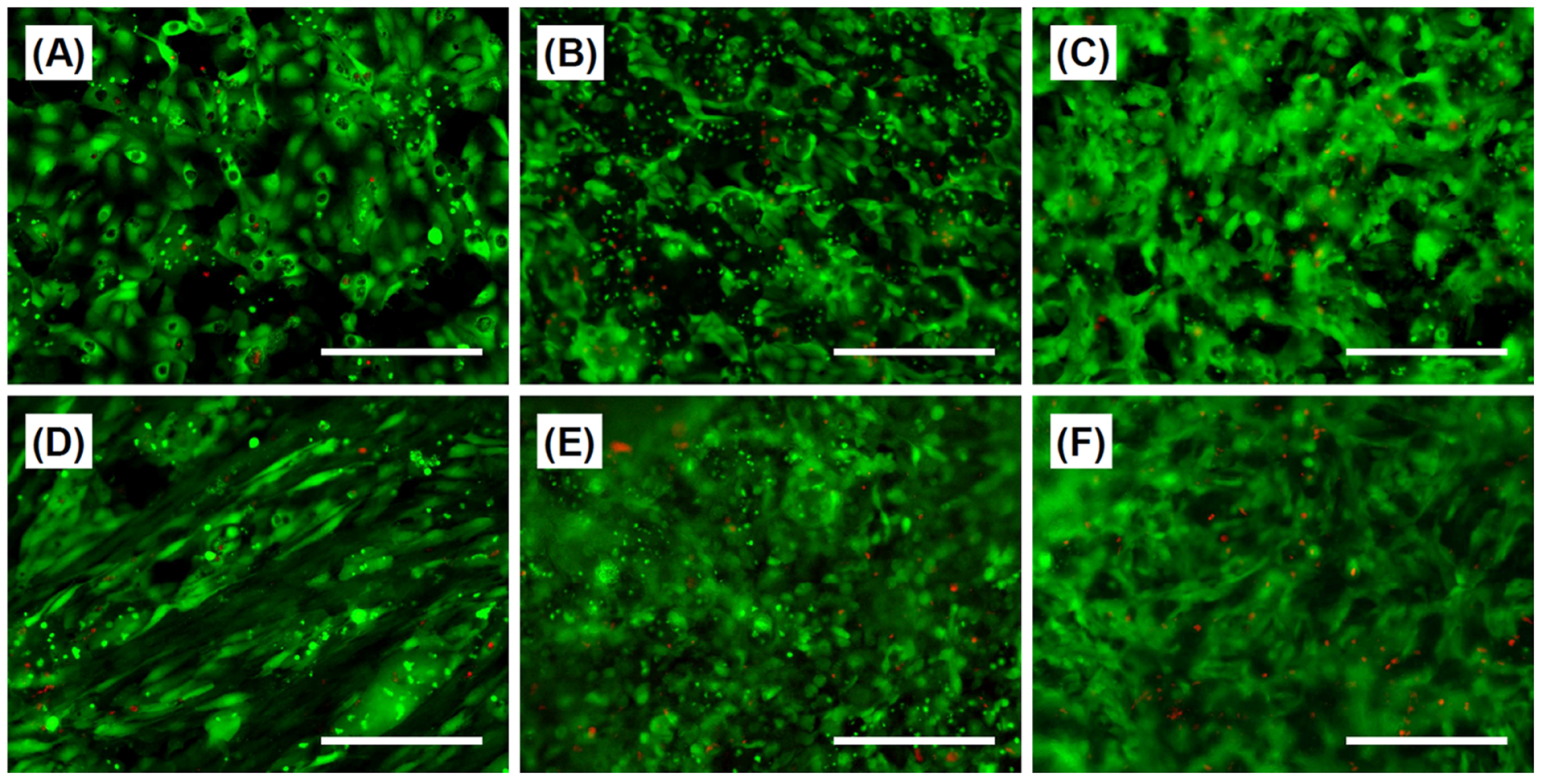
Live and dead stained fluorescent images. (A–C) HepaRG cells and (D–F) TIG-118/HepaRG cells. (A, D) After four days of culturing HepaRG cells on the TRCD and (B, E) after two hours and (C, F) 24 hours of reculturing cell sheets on glass-based dishes. Green (Calcein-AM): viable cells, red (PI): dead cells. The bar represents 200 µm.

Multi-layered cell sheets are required for the fabrication of 3D tissue and/or good handling in transplantation. The tissue size of the cell aggregates, including cell sheet and spheroids, is one of the most important topics related to cell survival, because the cell aggregates without capillary vessels are only supplied oxygen from surface diffusion. In this paper, almost all of the cells comprising cell sheets remained alive after shrinkage, even in the center of the laminated TIG-118/HepaRG cell sheets (Figs. 8A, 8B, 8D and 8E). In terms of the oxygen diffusion/supply, a cell sheet is thought to remain alive and healthy up to approximately 40 µm in thickness [27]–[30]. Therefore, TIG-118/HepaRG cell sheets may remain viable because their thickness is less than 40 µm. In addition, there was not significant increase in the number of dead cells in the TIG-118/HepaRG cell sheet after 24 hours of reculture in spite of the strong shrinkage of TIG-118 cells (Fig. 8F). Therefore, the TIG-118/HepaRG cell sheets produced in this study may be useful as a transplantable treatment tool, with favorable handling and engraftment properties.

### Liver-specific Functions

**Figure 9:**
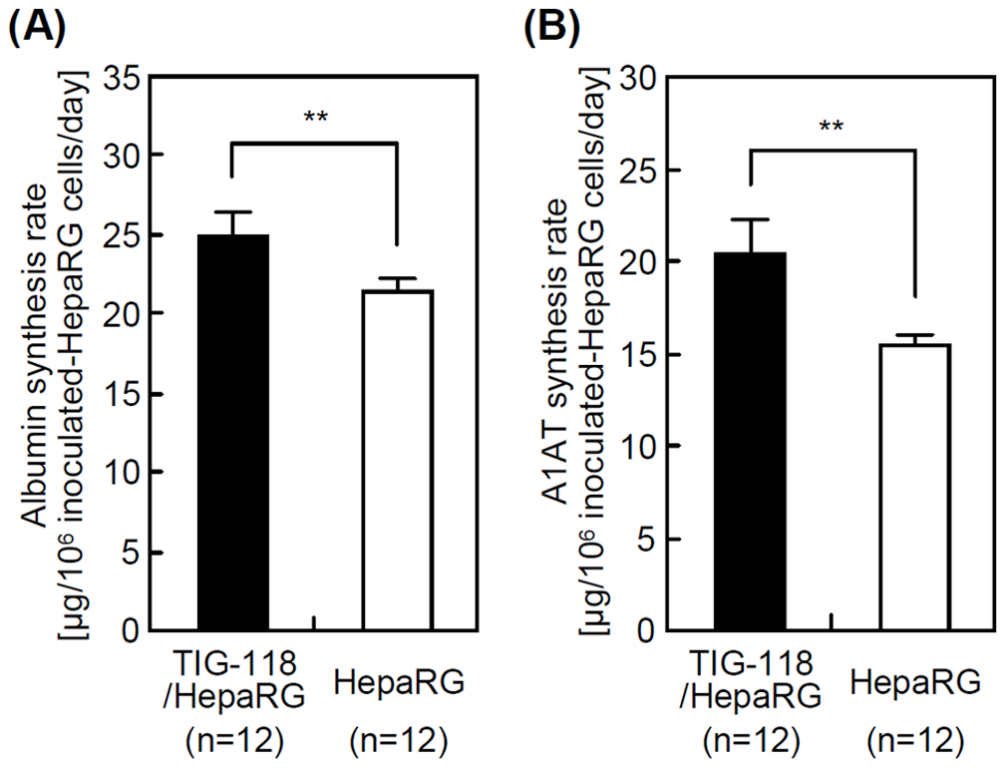
Liver-specific functions of HepaRG cells and TIG-118/HepaRG cells on TRCD. Human albumin (A) and A1AT (B) synthesis rates.

Figures 9A and 9B show the human albumin and A1AT synthesis rates of HepaRG cells and TIG-118/HepaRG cells on TRCD. The albumin and A1AT synthesis activities of TIG-118/HepaRG cells were approximately 1.2 and 1.3 times higher than those of the HepaRG cells, respectively. As reported in previous studies, this result is considered to be attributable to the stable adhesion of hepatic cells, formation of a large aggregate 3D colony such as spherical multicellular aggregates (spheroids), and the co-culture-based growth factor (including angiogenic factors from fibroblasts) or cytokine delivery [11], [12], [17]–[20], [23], [24]. In addition, abundant cell-cell adhesions will increase these functions.

In the future, a more detailed analysis is necessary to identify the secreted growth factors (e.g., hepatocyte growth factor, epidermal growth factor, fibroblast growth factor) responsible for the improvement of the liver-specific functions. In addition, evaluating angiogenic factors (e.g., platelet-derived growth factor and vascular endothelial growth factor) is also necessary in order to investigate the fabrication of 3D architecture and vasculature structure for *in vivo* survival. Although the liver-specific functions were only shown for the initial culture period in this paper, their long-term maintenance is expected. This idea supported by the fact that the HepaRG cells are a well-differentiated cell line that stably expresses high levels of liver-specific functions for least two months in culture [31], [32]. In the future, we will demonstrate these functions in our system by using primary hepatocytes.

### Conclusions

In this study, multi-layered human hepatic cell sheets were fabricated rapidly and efficiently by using fibroblasts as feeder cells. TIG-118/HepaRG cell sheets were thicker and easier to handle compared with cell sheets consisting of only HepaRG cells, and they had a favorable survival and expressed a high level of liver-specific functional markers. These results indicate significant advantages with respect to cell adhesion in hepatocyte transplantation and the handling properties of cell sheets.

Remarkably, this present technique focused on the forceful contraction of fibroblasts without requiring either cell proliferation, layer-by-layer deposition or culturing with a high cell density. Therefore, this technique is a promising avenue for the rapid and efficient fabrication of human hepatocyte sheets with low growth activity, and these cell sheets may be used for cell transplantation with highly specific functionality for regenerative medicine. Moreover, this technique can also be utilized for the fabrication of cell sheets for primary cells, including pancreatic islets with low cell proliferation potential which have a need for coating of suitable ECM associated with cell sheet fabrication.

Future research should be conducted concerning production of several growth factors and cell sheet transplantation (e.g., *in vivo* survival, fabrication of tissue and pathological hallmarks). At the same time, it is expected that these results are likely to vary significantly depending on the combination of different types of hepatic cells and the support cells, which is an interesting subject for further studies about transplantable treatment tools.
